# Plant Screen Mobile: an open-source mobile device app for plant trait analysis

**DOI:** 10.1186/s13007-019-0386-z

**Published:** 2019-01-11

**Authors:** Mark Müller-Linow, Jens Wilhelm, Christoph Briese, Tobias Wojciechowski, Ulrich Schurr, Fabio Fiorani

**Affiliations:** 1 IBG-2: Plant Sciences, Institute for Bio- and Geosciences, Forschungszentrum Jülich, 52425 Jülich, Germany; 2 Present Address: German Aerospace Center (DLR), Lilienthalplatz 7, 38108 Brunswick, Germany

**Keywords:** Plant image segmentation, Image analysis, Mobile application, Android, Machine learning, Projected leaf area

## Abstract

**Background:**

The development of leaf area is one of the fundamental variables to quantify plant growth and physiological function and is therefore widely used to characterize genotypes and their interaction with the environment. To date, analysis of leaf area often requires elaborate and destructive measurements or imaging-based methods accompanied by automation that may result in costly solutions. Consequently in recent years there is an increasing trend towards simple and affordable sensor solutions and methodologies. A major focus is currently on harnessing the potential of applications developed for smartphones that provide access to analysis tools to a wide user basis. However, most existing applications entail significant manual effort during data acquisition and analysis.

**Results:**

With the development of *Plant Screen Mobile* we provide a suitable smartphone solution for estimating digital proxies of leaf area and biomass in various imaging scenarios in the lab, greenhouse and in the field. To distinguish between plant tissue and background the core of the application comprises different classification approaches that can be parametrized by users delivering results on-the-fly. We demonstrate the practical applications of computing projected leaf area based on two case studies with *Eragrostis* and *Musa* plants. These studies showed highly significant correlations with destructive measurements of leaf area and biomass from both ground truth measurements and estimations from well-established screening systems.

**Conclusions:**

We show that a smartphone together with our analysis tool *Plant Screen Mobile* is a suitable platform for rapid quantification of leaf and shoot development of various plant architectures. Beyond the estimation of projected leaf area the app can also be used to quantify color and shape parameters of other plant material including seeds and flowers.

**Electronic supplementary material:**

The online version of this article (10.1186/s13007-019-0386-z) contains supplementary material, which is available to authorized users.

## Background

The evaluation of leaf area development addresses one of the most important issues in plant phenotyping, but poses one of the biggest challenges at the same time. This key parameter characterizes the interface between canopy and atmosphere, which regulates photosynthetic activity and transpiration processes and is used to monitor shoot growth and to model plant and environment interactions ([1] and references therein). The high diversity of canopy structures at different growth stages results in a number of technical problems, like measurable canopy size, perspective limitations depending on view-angles, and self-occlusions within the canopy. Accordingly, a variety of imaging-based methodological developments and commercial solutions emerged in the last years (for a good overview, see [1–3]). They include non-invasive and indirect methods, which make use of radiative transfer models [4] and which measure at different angles and levels within the canopy [5], direct non-invasive methods like hand-held scanners [2], and laser-scanning [6, 7]. Many of these methods entail specifically developed hardware and consequently potentially significant investment costs. Alternative approaches that are easy to implement and cost-efficient at the same time became increasingly important. A first step in this direction were camera-based applications that make use of new image analysis tools. However, most of the published methods using this methodology are still invasive, i.e. they require the detachment of the leaves from the plant stem [8–10]. Leaves are then placed on an imaging plane with a suitable background providing optimal contrast, often using a reference pattern for metric conversion of pixel data. Such approaches facilitate leaf segmentation and reduce perspective effects during image acquisition, resulting in sufficiently precise and accurate leaf area measurements of single leaves. For example, Rico-Garcia et al. [8] tested a Computer Aided Design (CAD)-based approach against their image processing suite in tomato and corn and obtained a maximum error of ~ 4% with a deviation of ~ 3%; the error was defined as the percentage of over- or under-estimated leaf area. However, at the same time these methodologies are usually restricted to smaller sample sizes, mainly due to the time-consuming process of leaf sampling and image acquisition. Many scientific questions require screening setups, where the growth of whole plants is monitored over an extended period of time starting at germination and seedling stages. Those are exactly the conditions, where non-invasive imaging approaches have very good opportunities for applications. At the same time the processing capabilities of mobile phones as well as the quality of built-in cameras improved drastically such that smartphones can now be used for various purposes ranging from documentation and classification of samples to quantitative analysis based on pixel and color information of digital pictures [11]. A number of beneficial features promote this current trend. Mobile phones do not replace just cameras, but provide capability to run the analysis tasks on-board, independent of additional hardware and other infrastructure like network connection (e.g., for upload and server-based analysis), or external power supply. However, the number of published mobile phone applications, specifically developed for plant phenotyping tasks, is still limited to specific cases. Intaravanne et al. [12, 13] developed two applications, which focus on the analysis of color properties of banana fruits and rice leaves to determine the ripeness and nitrogen content, respectively. In seed phenotyping smartphone applications were used for automated characterization of seed morphology in crops [14, 15]. Another study used a smartphone for automated berry counting in grapevine in the field [16]. Leaf Doctor [17] is a mobile application, which analyzes color images to quantify the severity of different diseases that result in visible color changes of the leaf surface. Confalonieri et al. [18] applied the radiative transfer model and estimated the gap fraction in rice plantations with a smartphone application that segments canopy from the sky.

With *Plant Screen Mobile* we contribute a new approach for Android-based smart phones, which uses both on-board camera and processing unit for the analysis of shoot and leaf images, in particular. *Plant Screen Mobile* provides a portfolio of segmentation approaches, which enable the user to detect the target plant even in the absence of a contrasting background and furthermore under various illumination conditions. It can make use of the internal device storage, does not require external processing time, and is therefore suitable for studies in growth chambers, greenhouses, and in the field, where access to a network or computers is not guaranteed. The application completes the analysis with the possibility of geometric calibration and extracting traits like projected leaf are (PLA) and shape parameters like object size and perimeter from the images. Furthermore we implemented genetic parameter optimization for color segmentation. We tested its applicability to several test cases. We present an evaluation of its performance by comparison to measurements with typical lab camera setups, based on destructive measurements.

## Implementation

**Fig. 1:**
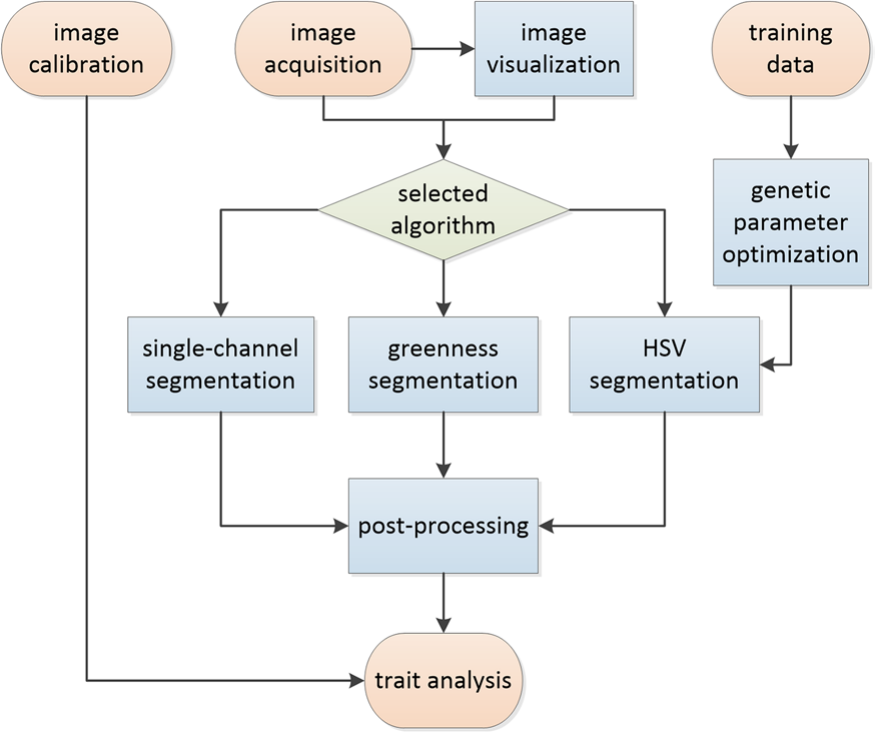
Flowchart of implemented processes: the main process (both on-the-fly and batch mode) start with the *image acquisition* as input and ends with analyzed traits. *Image calibration* allows to convert pixel metric into real-world values. Additionally, *training data* can be used to optimize parameters for HSV segmentation

*Plant Screen Mobile* (further denoted as PSM) was developed with Android Studio (Google Inc.) using the OpenCV-libraries for image processing and analysis tasks. It works on mobile phones with Android OS 4.0 (Ice Cream Sandwich). All computations were performed on a Samsung Galaxy S6 smartphone. An overview on the basic processing modes of PSM is illustrated in the flowchart in Fig. 1.

### Image acquisition and display

**Fig. 2:**
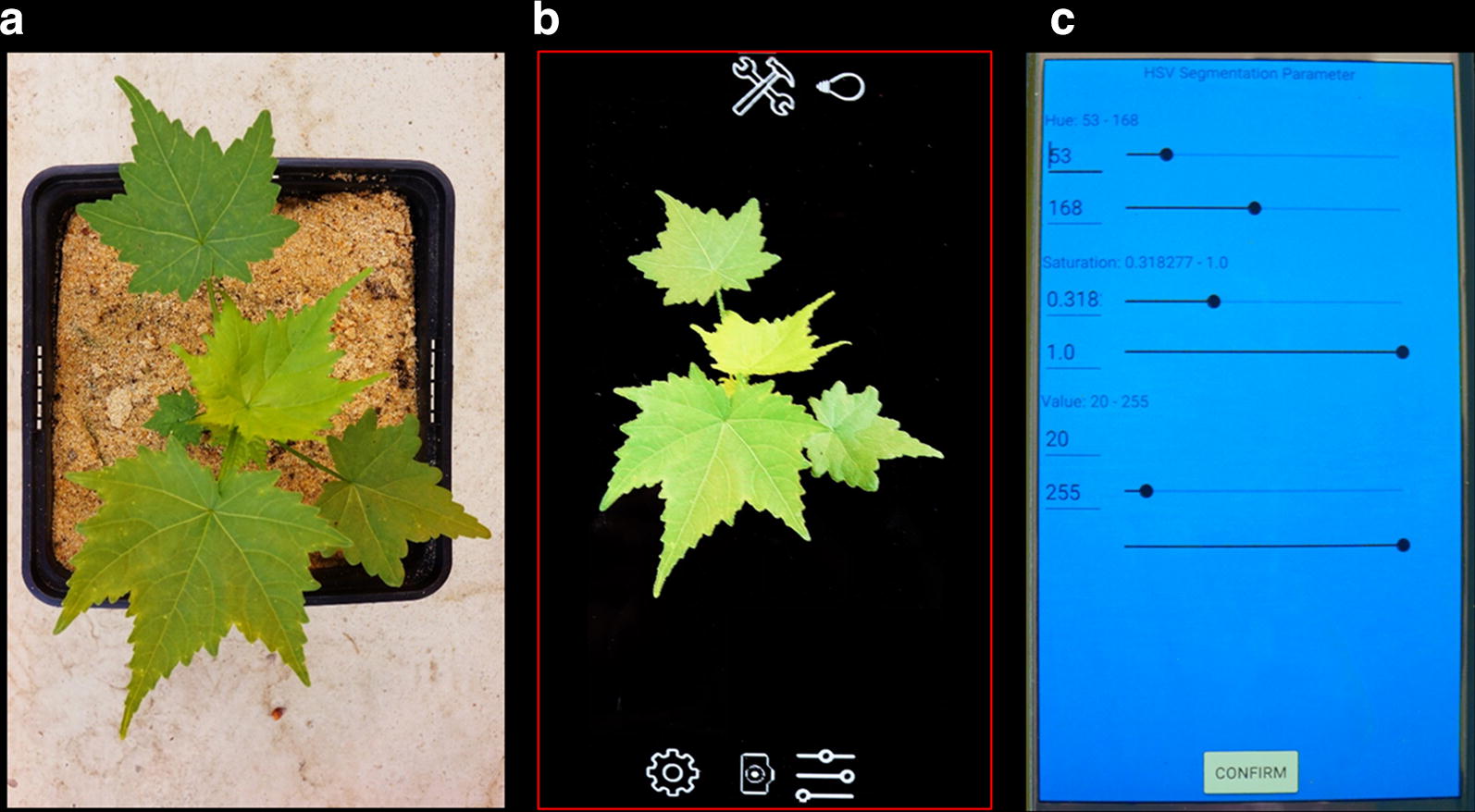
On-the-fly segmentation of a Virginia mallow plant (*Sida hermaphrodita*). **a** Image of a *Sida* plant; **b** live view image displaying the on-the-fly HSV-segmentation of the imaged seedling (live view image was taken from a slightly different angle); RGB-camera values are first converted into HSV color space and then binarized using the parametrization of the HSV-filter in **c** lower and upper thresholds of the HSV-filter are adjusted such that the resulting plant mask can be analyzed for projected leaf area and other plant traits

The main interface of *Plant Screen Mobile* displays a live image from the front camera at a maximum resolution of 1920 × 1080px (Fig. 2a). Images are either stored for later processing (e.g. to speed up image acquisition) or analyzed immediately with a given parameter set (Fig. 2b, c). In the latter case each implemented algorithm is directly applied to the live image, results are displayed on-the-fly and can be stored as masked images. In this mode no additional traits are computed. To reach the desired camera orientation PSM includes a level tool that uses the smartphone’s accelerometer. During adjustment tilt angles are continuously displayed and horizontal (top-view) or vertical (side-view) camera orientations are indicated as information on the live screen. If necessary the smartphone illumination headlight may be switched on to optimize capturing conditions. Different visualizations like single channel display in RGB (red, green, blue color space) or HSV (hue, saturation, value color space) and a color information tool for the screen center pixels help to judge the imaging situation and to parameterize the filters. If images are analyzed at a different time from acquisition or imported from other sources, PSM provides batch operation for multiple images with one of the pre-selected processing modes that will be explained in the following together with a pre-specified parameter set. Segmented image results are stored and computed traits, evaluation date, and time are exported to CSV files.

### Image segmentation

We implemented three image segmentation methods based on single channel thresholding (i), greenness thresholding (ii) and HSV-thresholding (iii) that deliver binary outputs to mask the image background. Selecting a suitable method depends on different factors. First of all, the image capture conditions determine which method is applicable or not. In the absence of color information, e.g. when imaging roots, the lightness is the main classifier and simple gray value thresholding should be sufficient. This method requires less computational power, but tends to lower accuracy in imaging situations with unequal illumination. If leaves are imaged under controlled conditions and classification is not disturbed other image ‘contaminants’ like algae or weed, greenness thresholding would be the method of choice. Requiring only one thresholding value it is far easier to parametrize, compared e.g. to HSV thresholding, which needs 6 parameters. Albeit higher computational costs, the last approach is suitable in more difficult imaging situations, were additional classifiers like saturation or lightness are needed. We tested the principal segmentation capabilities and performance differences between greenness and HSV thresholding and highlighted results in the Additional file 1.

(i) *Single-channel thresholding* When color is not the primary feature to distinguish between object and background, image segmentation via single-channel thresholding is a suitable method with respect to computational time and memory. We implemented three threshold operations that are employed on grayscale representation of the RGB color space: simple thresholding, adaptive thresholding, and OTSU’s method [19]. In simple thresholding intensity values of each image pixel are compared to a global threshold *α* resulting in a binary mask *B* with values of 1 indicating intensity values above *α* and 0 otherwise. These values are attributed to plant and non-plant pixels. This can be sufficient, if plants are homogeneously illuminated in front of a black background. In adaptive thresholding, which accounts for local variations in illumination, *α* is calculated separately for each pixel using the surrounding region of a preset size. The comparison is either based on averaged intensities (adaptive mean), or on the Gaussian weighted sum (adaptive Gaussian). In Otsu’s method threshold *α* is automatically calculated and applied to the entire image. The integral part is an estimation of *α*, which splits up the intensity distribution such that resultant distributions display low intra-variance and high inter-variance. If necessary, the image can be inverted before applying any threshold operation, e.g. to segment dark objects in front of a brighter background.
(ii) *Greenness thresholding* Many plant phenotyping applications require the segmentation of green plant tissue. Various suitable approaches with low computational costs have been introduced and tested in different application scenarios that compute greenness indices on the base of RGB channel intensities $I_{R } I_{G}$, and $I_{B}$ [20, 21]. We implemented three well-known greenness measures: the Green Chromatic Coordinate (GCC) [20], the Vegetative Index (VEG) [22] and the Excess Green Excess Red Index (ExGR) [23]. All indices can filtered by a single thresholding operation with parameter*α*. In these greenness definitions a pixel $I\left( {x,y} \right)$ at position (*x y*) is classified to *B* according to:
  $${\text{GCC:}}\quad B\left( {x,y} \right) = \left\{ {\begin{array}{*{20}l} {1\quad } \hfill & {if\quad \frac{{I_{G} \left( {x,y} \right)}}{{I_{R} \left( {x,y} \right) + I_{G} \left( {x,y} \right) + I_{B} \left( {x,y} \right)}} > \alpha } \hfill \\ {0\quad } \hfill & {otherwise} \hfill \\ \end{array} } \right.$$
  $${\text{VEG:}}\quad B\left( {x,y} \right) = \left\{ {\begin{array}{*{20}l} {1\quad } \hfill & {if\quad \frac{{I_{G} \left( {x,y} \right)}}{{I_{R} \left( {x,y} \right)^{{2/3}} *I_{B} \left( {x,y} \right)^{{1/3}} }} > \alpha } \hfill \\ {0\quad } \hfill & {otherwise} \hfill \\ \end{array} } \right.$$
  $${\text{ExGR:}}\quad B\left( {x,y} \right) = \left\{ {\begin{array}{*{20}l} {1\quad } \hfill & {if\quad 3I_{G}^{\prime } \left( {x,y} \right) - 2.4I_{R}^{\prime } \left( {x,y} \right) - I_{B}^{\prime } \left( {x,y} \right) > \alpha } \hfill \\ {0\quad } \hfill & {otherwise} \hfill \\ \end{array} } \right.$$
  with$$I_{R}^{{\prime }} \left( {x,y} \right) = \frac{{I_{R} \left( {x,y} \right)}}{{I_{R} \left( {x,y} \right) + I_{G} \left( {x,y} \right) + I_{B} \left( {x,y} \right)}};$$ $$I_{G}^{{\prime }} \left( {x,y} \right) = \frac{{I_{G} \left( {x,y} \right)}}{{I_{R} \left( {x,y} \right) + I_{G} \left( {x,y} \right) + I_{B} \left( {x,y} \right)}};$$ $$I_{R}^{{\prime }} \left( {x,y} \right) = \frac{{I_{B} \left( {x,y} \right)}}{{I_{R} \left( {x,y} \right) + I_{G} \left( {x,y} \right) + I_{B} \left( {x,y} \right)}}.$$

Despite their name, greenness filters are not highly sensitive to green colors only but also to the adjacent colors in the spectrum. In the presence of both blue-green and yellow-green colors these filters are rather unspecific and parametrization of alpha becomes increasingly difficult. In these cases we find that the HSV filter is a good alternative.

(iii) *HSV thresholding* This segmentation approach is widely used and well established in plant phenotyping (for details see e.g. [24]). It usually outperforms RGB-based segmentations, when color is the key feature of interest. In the HSV color space Hue (H) is associated with the dominant wavelength of captured light, saturation (S) is inversely proportional to the amount of white light mixed with hue, while value (V) is given by the maximum radiance in all RGB color channels. Because color is only represented by Hue, thresholding operations are far easier to apply and to adapt to different segmentation tasks. HSV thresholding requires a conversion from RGB to HSV color space. After that, thresholding operations are applied as follows with defined ranges for each channel in *I*:
  $$B\left( {x,y} \right) = \left\{ {\begin{array}{*{20}l} 1 \hfill & {{\text{if}}\quad \begin{array}{*{20}l} {H_{{min}} < I_{H} \left( {x,y} \right) < H_{{max}} } \hfill \\ {S_{{min}} < I_{S} \left( {x,y} \right) < S_{{max}} } \hfill \\ {V_{{min}} < I_{V} \left( {x,y} \right) < V_{{max}} } \hfill \\ \end{array} } \hfill \\ 0 \hfill & {otherwise} \hfill \\ \end{array} } \right.$$

### HSV parameter optimization via a genetic algorithm

To optimize HSV-segmentation results the PSM app computes suitable parameter sets from example images using a genetic algorithm for parameter optimization [25, 26]. This supervised approach requires at least one training image together with the ground truth, which is a binary image, where the target object (e.g. plant pixels) are labelled with ones and background pixels with zeros. PSM does not contain tools to produce ground truth images, but other software is suitable for this task. We recommend to use a computer or tablet with a larger screen for this purpose. The genetic algorithm starts with a population of *k* potential solutions (individuals), each of them with a set of parameters *G* (genome). During the iterative optimization process (*i* generations) the genome is altered by bio-inspired operations such as mutation, crossing and selection. In our case, the genome G consists of 6 thresholds:

$$G = ( H_{min} |H_{max} |S_{min} |S_{max} |V_{min} |V_{max} )$$

The quality of optimization is evaluated via a fitness function *f*, which needs to be customized to the problem and parameter set. Here, *f* is defined as the percentage of correctly classified values.

The genetic optimization algorithm consists of the following steps:

- *Step 1 *create a start population of k individuals with random values in the ranges of H, S and V.
- *Step 2 *calculate fitness f for each individual.
- *Step 3 *select the *n*-best individuals for reproduction.
- *Step 4 *create new offspring from every combination from the *n*-best individuals with crossing overs at probability P_*C*_ (crossing over rate).
- *Step 5 *mutate each gene of each individual from the genepool with probability P_*m*_ (mutation rate) and random value within range M (mutation range); continue with Step 2.

**Fig. 3:**
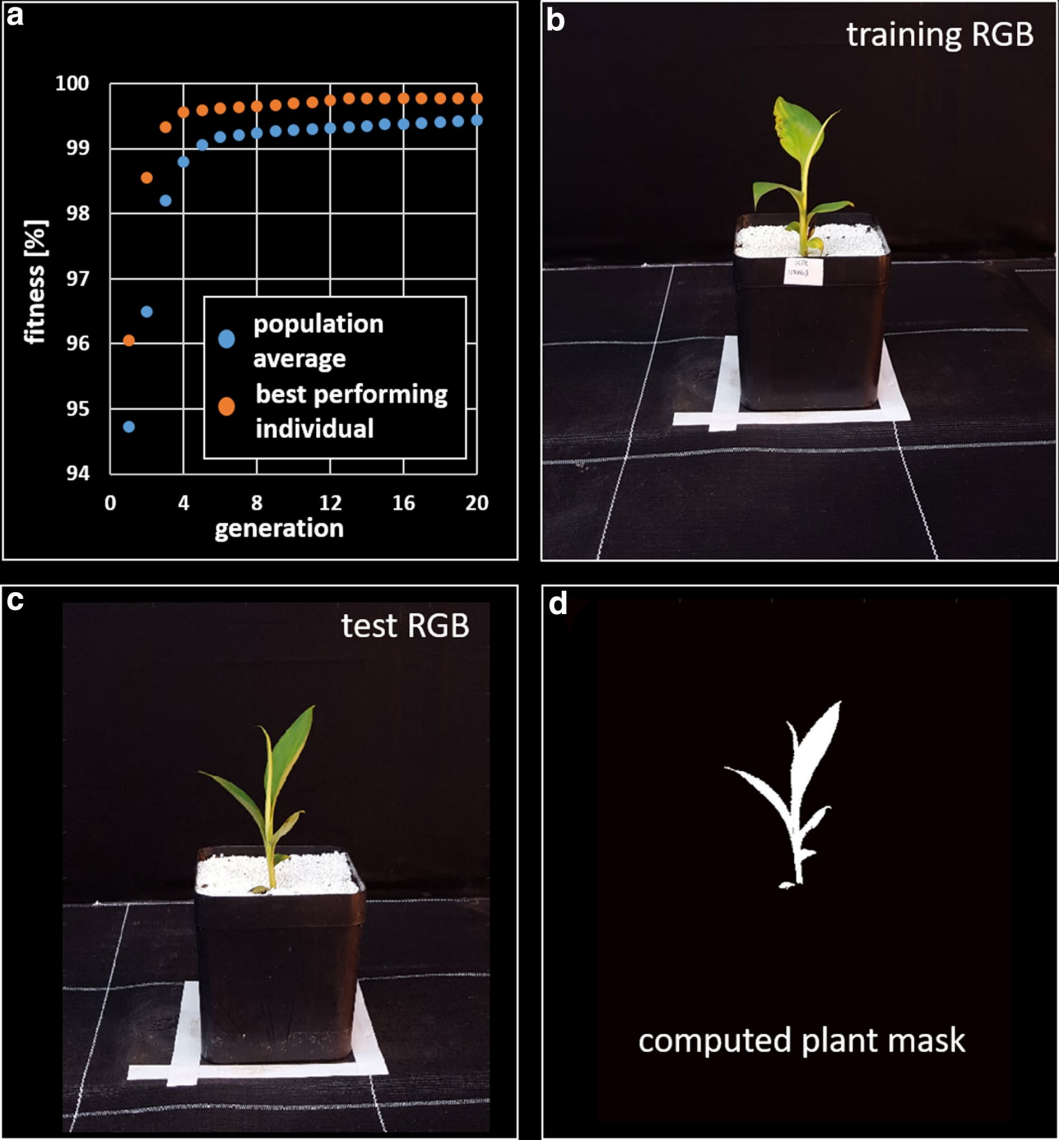
Genetic optimization of HSV thresholding parameters: **a** Illustration of the fitness function for the whole population (blue) and the best performing individual (red); after 3 generations more than 99% of the pixels are classified correctly (99.75% after 12 generations). **b** Training image, which served as the input for the optimization process; **c** test image and **d** corresponding test image mask that was computed with the optimized HSV parameter set

We tested this approach with sample images from the banana dataset (see “Results”). We split images into training and validation sets, each with 40 samples. We acquired ground truth image masks for both sets via HSV segmentation with individual parameter sets for each image and subsequent manual correction of the labels. In the genetic algorithm we choose a population of $k = 20$ individuals at $i = 250$ generations with a mutation rate of $P_{M} = 0.1$ and a mutation range of $M = \pm 1\%$ (of each respective channel range). The $n = 5$ individuals with highest fitness scores *f* were crossed with each other with $P_{C} = 0.5$ creating new individuals for the next generation. To determine the best configuration for *G* we processes all training images in one turn, repeated this process 20 times and computed the average fitness function $\bar{f}$ of the best performing individual. Figure 3a shows the performance of the best performing individual in comparison to the average performance of the entire population for the first 20 generations. After 250 generations the fitness reached a value of $\bar{f} = 99.87 \pm 0.02\%$ (no substantial progress $\bar{f}$ could be perceived). Already after 12 generations the best performing individual reached a fitness of $\bar{f} = 99.75 \%$. In the next step the best performing genome *G* was used to segment the validation images and resulting image masks were compared with ground truth by computing once more the fitness function *f*. The fitness for all 40 validation images averaged $\bar{f} = 99.84 \pm 0.05 \%$. In Fig. 3 we show sample images that was used for training (Fig. 3b), for testing (Fig. 3c) and the final plant image mask (Fig. 3d). As this optimization process requires a substantial amount of time, we recommend not to use more than 25 iterations. As illustrated above $\bar{f}$ will not improve notably after a few iterations. On a Samsung Galaxy S6 the processing time was approximately 20 min for 25 generations.

### Image post-processing and analysis

Each computed segmentation is post-processed in two steps. First, morphological operations (erosion and dilation) are applied to close small gaps and to remove small fragments. Then, components with an 8-connected neighborhood are identified and labeled. In this way, multiple objects like detached leaves are counted and analyzed at the same time. The estimation of projected leaf area is the key feature of PSM and the output are pixels counts for each segment. To ensure comparability between different measurements (e.g. in setups with a varying camera-to-plant distance) PSM allows for a pixel-to-area conversion. For this purpose the user needs to place a calibration target (e.g., checkerboard pattern [27]) at approximately the same distance as the plant object to be photographed. PSM automatically detects the pattern and calculates a conversion factor that is used to compute metric area values from pixel counts. Besides the estimation of projected leaf area, PSM also provides a number of additional measures, which are listed in Table 1. Analyzed traits are exported to a CSV file together with information on luminance (LUX) and GPS coordinates.

**Table 1:** Estimated Traits (for entire image or single segments): if analysis of single segments is enabled PSM delivers the number of segments (e.g. can be used for object counting) and the statistics on each single segment

Trait	Definition	Unit
Projected leaf area (PLA)	Segment-wise pixel sum	px/mm^2^
Perimeter	Length of segment contour	px
Segment width	Maximum horizontal segment stretch	px/mm
segment height	Maximum vertical segment stretch	px/mm
Red mean	Average intensity in the red channel	Channel intensity
Green mean	Average intensity in the green channel	Channel intensity
Blue mean	Average intensity in the blue channel	Channel intensity
Hue mean	Average intensity in the hue channel	Channel intensity
Saturation mean	Average intensity in the saturation channel	Channel intensity
Value mean	Average intensity in the value channel	Channel intensity

## Results

We tested *Plant Screen Mobile* in two different application scenarios to evaluate both versatility and performance. In these case studies we examined plants with contrasting shoot architecture, banana and both *Eragrostis tef* and *Eragrostis pilosa.* Two genotypes of banana plantlets were obtained from University of Hohenheim–Institute of Crop Science (Crop Physiology of Specialty Crops), Germany. Khai Thong Ruang KTR (Musa AAA) is a drought-sensitive desert banana from Thailand, Saba (Musa ABB) is a drought-tolerant African plantain. In total we used 52 replicates, 27 KTR and 25 Saba. In the *Eragrostis* experiment we used two species, i.e. 100 replicates in *Eragrostis tef* (teff) and 40 replicates in *Eragrostis pilosa*. Teff is a monocotyledonous species used in many parts of Africa, India, Australia and northern America. *Eragrostis pilosa* has no economic significance. The examples in the Additional file 2 show typical images of banana and *Eragrostis* that were used in our experiments. For the destructive measurements plant leaves where weighed with a high-accuracy lab balance (XS 205, Mettler Toledo, United States) and measured with a leaf area meter (LI-3100, Licor, United States) to obtain the true leaf area destructively.

Each plant was imaged from 4 sides adding up to 208 images in banana and 560 images in *Eragrostis*. Projected leaf area was estimated with PSM and compared against SVM-classified images that were acquired and analyzed with the SCREENHOUSE imaging system of IBG-2, Forschungszentrum Jülich GmbH [28]. The SCREENHOUSE is an automated greenhouse plant phenotyping platform, equipped with an imaging station for data acquisition under controlled light conditions. It is equipped with three RGB cameras (Grasshopper 2, Point Grey Research, 5MP) that image plants from three different view angles. Support vector machine (SVM) classification [29] of foreground and background pixels is a supervised approach based on training data sets, which generally yields very good solutions for linear- and nonlinear separable data regarding stability and accuracy and which is robust against outliers in the data. In both approaches the projected leaf area of each plant was averaged over 4 views, in the case of PSM outside the application using the csv output file and MS Excel.

### Projected leaf area of *Eragrostris* and banana whole plants

**Fig. 4:**
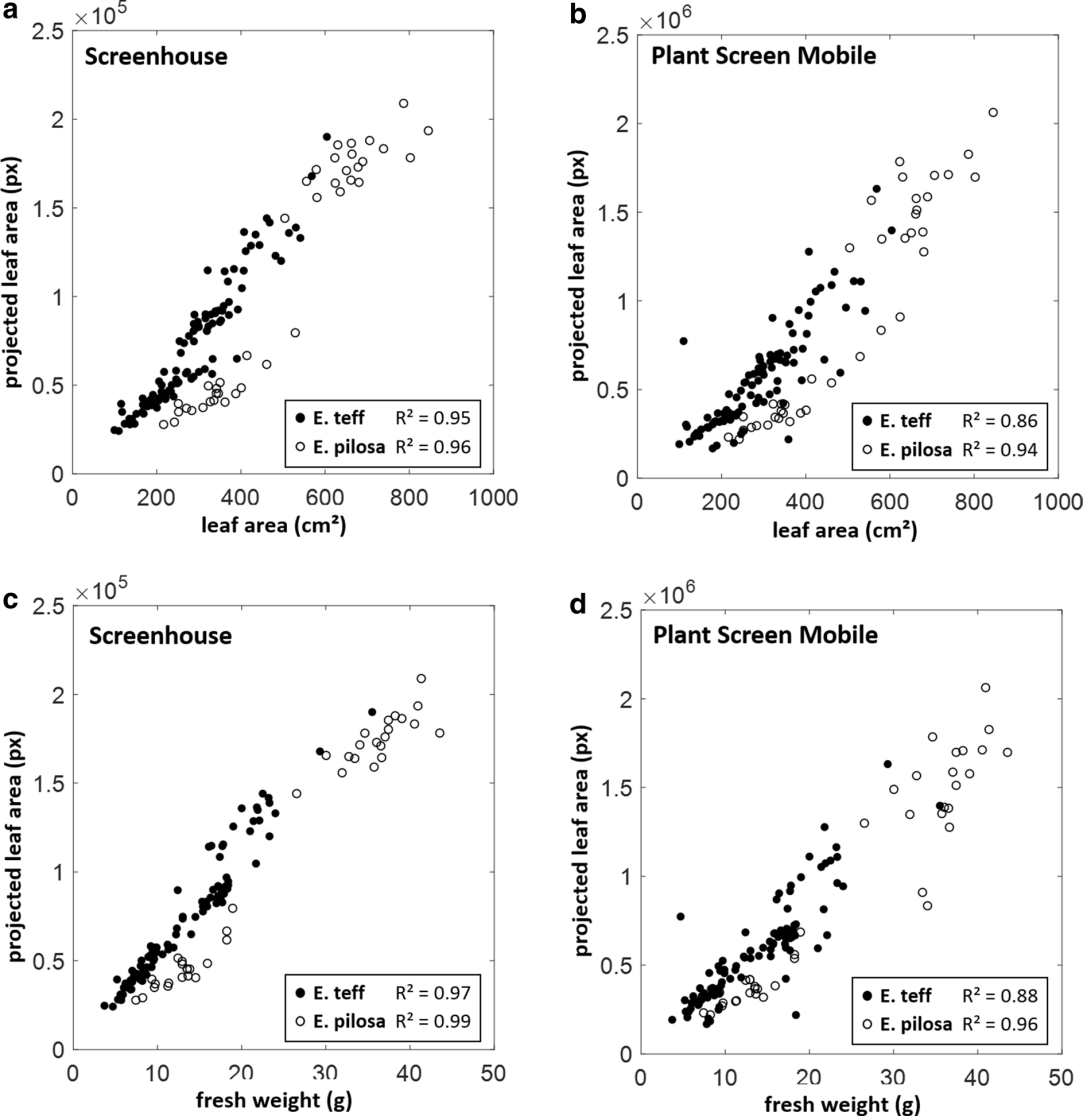
Case study *Eragrostis*: Projected leaf area (PLA) of complete plants of two *Eragrostis* species estimated with the Screenhouse System (**a**, **c**) and *Plant Screen Mobile* application (**b**, **d**) was compared to measured leaf area (**a**, **b**) and measured fresh weight (**c**, **d**)

**Fig. 5:**
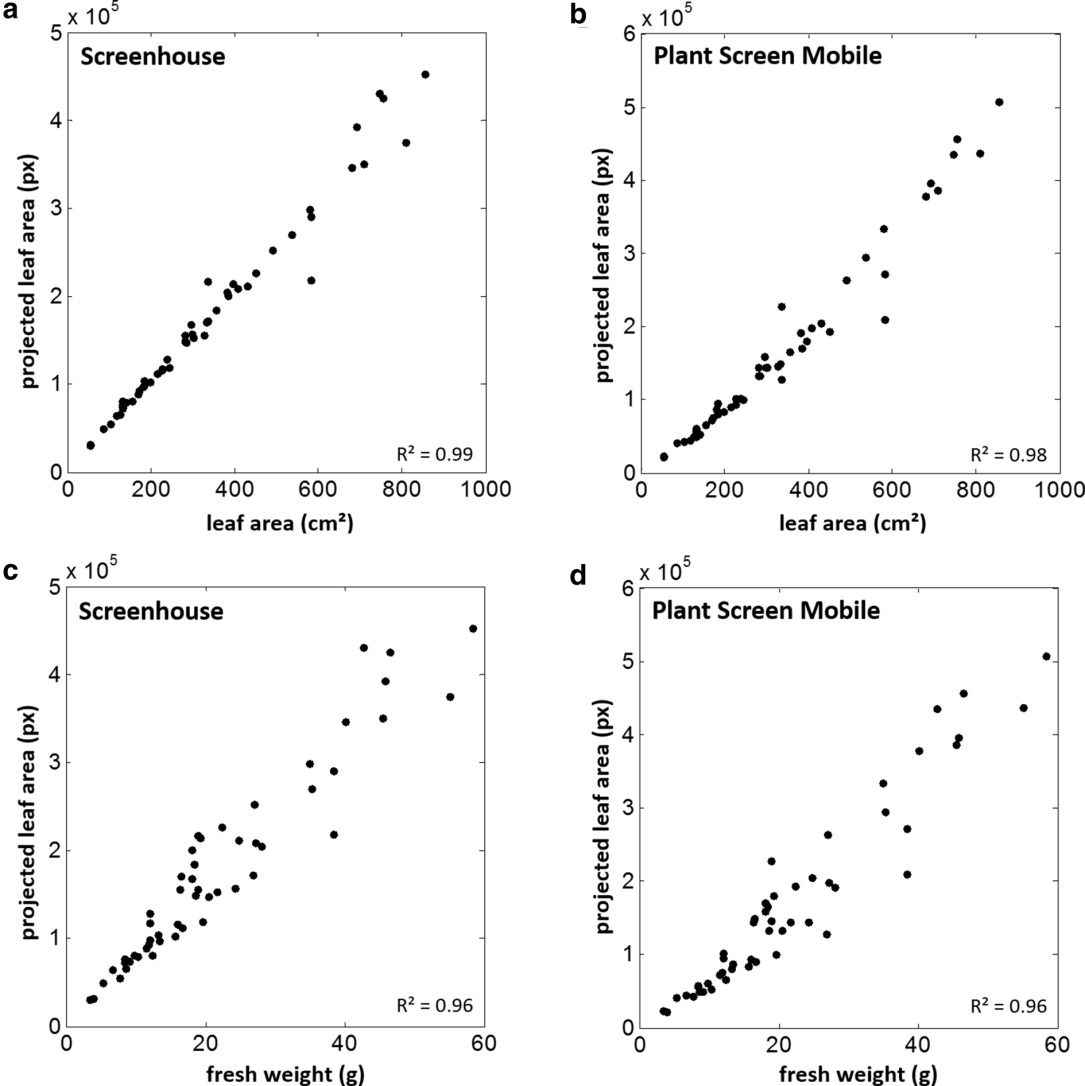
Case study banana: Projected leaf area (PLA) of complete banana plants estimated with the SCREENHOUSE System (**a**, **c**) and *Plant Screen Mobile* application (**b**, **d**) was compared to measured leaf area (**a**, **b**) and measured fresh weight (**c**, **d**)

In these two case studies, we compared estimations of projected leaf area of *Eragrostris tef* and *pilosa* plants as well as banana plants, which were imaged with a smartphone (Galaxy S6, Samsung Electronics) and similarly with the imaging system of the SCREENHOUSE. In our test scenario, we used a side view perspective (perpendicular to the shoot axis). In order to obtain a comparable imaging setup, the smartphone was fixed on a tripod at about the same distance and orientation as the corresponding side view SCREENHOUSE camera. Camera settings were IS0 100, exposure compensation − 2.0, white balance 5500 K and manual focus without flash in both experiments. To take advantage of the highest camera resolution thereby speeding up image acquisition, images were captured successively and analyzed afterwards with the batch processing option of PSM. All images were segmented with the greenness thresholding method using the ExGR Index with $\alpha = - \,0.03$ for *Eragrostis* and $\alpha = 0.03$ for banana plants. Images from the SCREENHOUSE were analyzed with our in-house segmentation software, which uses a pre-trained classifier based on features from the three RGB channels and a polynomial SVM kernel, which showed best results in our test scenarios. Plant fresh weight and leaf area was measured destructively. Projected leaf area estimated by PSM shows high correlations to both reference measurements (weight and leaf area, Figs. 4, 5). Taking the differences between *R*^2^-values as an indicator PSM performs only slightly worse in comparison to the SCREENHOUSE imaging system. In banana and *E. pilosa* there were no remarkable effects. In *E. pilosa* $\Delta R^{2}$ was 0.02 for LA and 0.03 for weight. The difference in *E. tef* was slightly more pronounced with values of $\Delta R^{2} = 0.09$ for both LA and weight. In the *Eragrostis* case study (Fig. 4) both species *E. tef* and *E. pilosa* differ in leaf architecture and could therefore be distinguished in the diagrams. In comparison to *E. tef* the more dense leaf display of *E. pilosa* results in lower PLA estimations especially for smaller plants. Leaf area and fresh weight results are comparable, which could be taken as an indicator for minor variations in leaf thickness. Lower *R*^2^-values in *E. tef* for PSM are mainly caused by a few data points that are most likely underestimated particularly in comparison to the SCREENHOUSE results. In the evaluation of the banana experiment we did not distinguish between both genotypes as they displayed no significant difference. Therefore both results are combined in Fig. 5. PSM results differ only marginally from the SCREENHOUSE evaluation making this plant particularly suitable for the analysis with the mobile application at the developmental stages afforded by this study.

In the Additional file 2 we compiled various examples to display additional application scenarios; the first two show typical shoot images of *Eragrostis* and banana. In the following we tested the app also with root images (*Cassava*), blossom segmentation and seed segmentation (here: barley and rape seeds).

## Discussion

The segmentation process is the crucial step towards the quantitative estimation of leaf area or other plant traits. Therefore, we implemented a range of different approaches that use channel intensities in Gray, RGB or HSV color space. All these methods have in common that they can be processed on Android-based smartphones (of the last generations). We tested the smartphone App on African plant species with contrasting leaf architecture to showcase their application as an affordable phenotyping device supporting research where larger investments may be prohibited. In a controlled setup we could show that differences between PSM and an established shoot imaging platform like our SCREENHOUSE that uses powerful SVM segmentation are marginal. Banana and *Eragrostis pilosa* plants displayed barely any difference, while the difference ($\Delta R^{2} = 0.09$) in *Eragrostis tef* plants was the largest. Most likely these deviations are not a consequence of different hardware. Differences rather emerge from the applied processing method. Here, the SVM method benefits from its better classification capabilities. In PSM the PLA of a few *Eragrostis tef* plants was underestimated leading to a weaker correlation (Fig. 4). It must therefore be assumed that the implemented PSM methods work equally well if foreground and background features (color, intensities) are clearly separable. If this is not the case, e.g. if plants grow in the presence of other weeds or algae develop on the substrate surface, more sophisticated methods like the presented SVM method or likewise methods (e.g. Random Forest classifiers) may help. However, one has to consider the many cases, especially in outdoor applications, where training data has to be produced again and again to account for changing imaging conditions. Additional training time and computational costs would arise that can hardly be managed efficiently on a smartphone. Here the advantages of a more flexible application like PSM are evident, where the outcome can be visually controlled and changed by simple re-adjustment of parameters. We displayed the application in a robust setup, where PSM can be used in batch processing mode together with genetic parameter optimization and a pixel-to-metric system calibration. If used in less controlled application scenarios (e.g. outdoors with changing illumination) each imaging situation can be parameterized individually in order to achieve comparable results. We also highlight that PSM is not restricted to the detection of shoot and leaves. The HSV filter offers enough opportunities also to detect other plant parts like blossoms, fruits or seeds.

## Conclusion

Estimation of plant traits by digital imaging, which requires the segmentation of imaged objects (plants and leaves as well as blossoms, fruits or seeds) is still one of the biggest challenge in non-invasive plant phenotyping approaches. There are numerous methods, which have been introduced in recent times, however for mobile solutions such as smartphones or other handheld devices there is still a lack of applications, especially ones that are generic and that make use of smartphone processing power and combine other sensors data (e.g., GPS). Constraints are not only given by the lower processing capabilities, which e.g. limit the use of machine learning based methodologies, but also the evident usage of such devices in various application scenarios that range from controlled imaging conditions (like a lab or dedicated imaging box) to not-controlled conditions (greenhouse or outdoor). With the development of *Plant Screen Mobile* we provide a new analysis tool that exploits various smartphone capabilities to easily quantify contrasting leaf architectures with respect to projected leaf area, which can be used as proxy for leaf area and biomass. The central processing step is the separation of plants from imaged background. Here we included different solutions that can be selected according to image acquisition conditions and desired processing speed, which also determines the speed of visual feedback for the user. The image stream is processed on-the-fly and thus the user is able to parametrize the analysis effectively and rapidly. Furthermore, we explored different application scenarios and we conclude that PSM is sufficiently versatile for a variety of plant tissues and illumination conditions.

## Additional files

**Additional file 1.** Performance comparison between greenness and HSV segmentation.

**Additional file 2.** Application Scenarios for Plant Screen Mobile.

## Abbreviations

*α*: threshold that is applied on an image to separate plant from background pixels resulting in a binary mask *B*
*B*: binary image of ones (plant pixels) and zeros (background pixels)
CSV: data file of comma separated values
ExGR: *Excess Green Excess Red* greenness index
*f*: fitness value in the genetic algorithm
*G*: genome in a genetic algorithm
GCC: *Green Chromatic Coordinate* greenness index
H, S, and V: hue, saturation, and value channels of the HSV color space that result from a transformation of RGB color space
*I*_*R*_*I*_*G*_, and *I*_*B*_: intensity values of each RBG channel
*I*_*H*_*I*_*S*_, and *I*_*V*_: intensity values of each HSV channel
*i*: number of generations in the genetic algorithm
*k*: number of potential solutions (individuals) in the genetic algorithm
*M*: mutation range
*P*_*C*_: crossing over rate
*P*_*M*_: mutation rate
PSM: Plant Screen Mobile application for smartphones
R, G, and B: red, green, and blue channels of the an RGB image
SVM: support vector machine
VEG: *Vegetative* greenness index
x, y: pixel position in an image at x (column index) and y (row index)
