# Supplementary material for: Plant Screen Mobile: an open-source mobile device app for plant trait analysis
Source: Plant Methods. 2019 Jan 11;15:2. doi: 10.1186/s13007-019-0386-z (PMC6329080; doi:10.1186/s13007-019-0386-z)

**Additional file 1: Performance comparison between greenness and HSV segmentation**

We performed two tests to display performance differences between greenness and HSV segmentation with respect to precision and accuracy. The first test is based on the two data sets presented in the main part, which are images of Banana and *Eragrostis* plants. Projected leaf area results from both HSV and greenness thresholding were compared against ground truth values from leaf area meter measurements. The second test (see Figure) uses an image of a seedling to showcase the capabilities and restrictions of both segmentation approaches.

*Test 1:* In the main part of this manuscript we already presented two test cases, which were analyzed with the greenness segmentation approach. Now, we compare these results against segmentations from HSV thresholding. To find an optimal parametrization of the HSV filter, we applied the genetic algorithm (as explained in the Implementation) on the entire plant sets of Banana, *Eragrostis* *pilosa* and *Eragrostis teff*, using one image from the four turn table views as input data. Corresponding SVM segmentations from the Screenhouse Imaging System were used as reference during the optimization process. Resulting HSV thresholding parameters were then applied on the complete image data sets and projected plant leaf area (PLA) was averaged over 4 views. We compared PLA with ground truth LA values (from leaf area meter measurements) and computed the coefficient of determination R² as performance indicator for estimation precision. In the banana experiment both greenness and HSV method resulted in R² = 0.98. More pronounced differences could be observed in the *Eragrostis* experiment. *E. pilosa* displayed R² = 0.94 (greenness) and R² = 0.97 (HSV), while *E. teff* reached R² = 0.86 (greenness) and 0.9 (HSV). As expected HSV segmentation allowed for a slightly better classification. However, as images were acquired under the controlled conditions in a lab setup, no marked differences could be expected.

*Test 2:* For this test an image of a single seedling planted in a tray was chosen (A). The cultivation soil was covered by a dark green moss layer and therefore it was expected that the image analysis would be complicated. Ground truth was produced manually with MS paint (B) and served as reference for accuracy measurements. The best possible seedling segmentation via greenness thresholding was achieved by first computing the ExGR greenness index (C), here rescaled to values between 0 and 255, and then applying thresholds in the same range. Each resulting segmentation was compared against ground truth by computing a classification error E_I_, which is the percentage of misclassified pixels of seedling and background (D). The highest accuracy was achieved at a minimum E_I_ = 4.7 % and the corresponding threshold was used to compute the segmentation in (E). Thresholding parameters for the best possible HSV segmentation were again estimated via the genetic algorithm using the ground truth image in (B) as reference. Compared to greenness thresholding the output displays a considerably improved classification and accuracy (F) with an E_I_ = 1 %. The seedling itself displayed 40.2 % of misclassified pixels for greenness and 5.8 % for HSV thresholding, respectively. This is a nice example to demonstrate the restrictions of the greenness method. In the presence of the green moss layer (even if it is such dark and therefore easy to distinguish by eye) this approach fails, because additional classification criteria like lightness or saturation are missing.


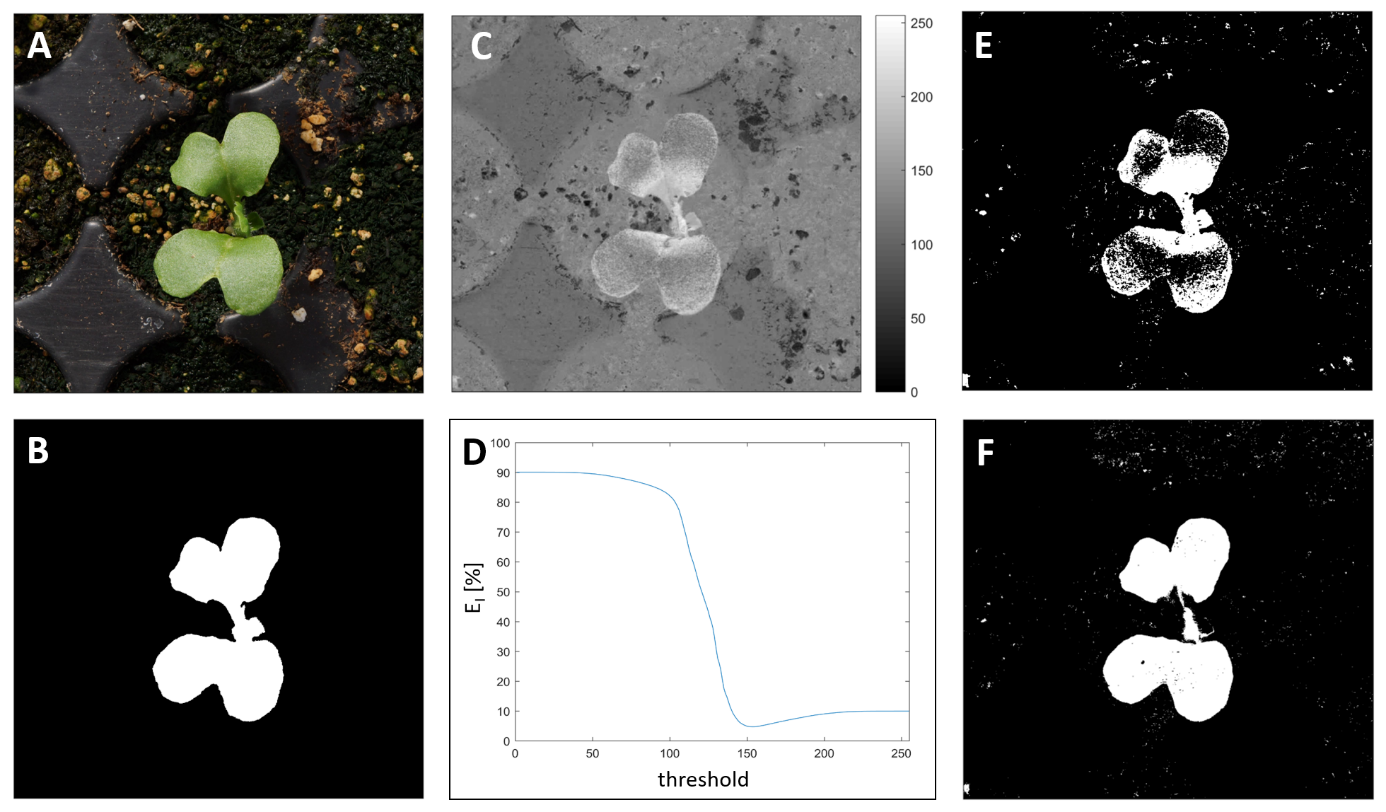

Supplement: Supplementary file 1 — Additional file 1. Performance comparison between greenness and HSV segmentation. [file 13007_2019_386_MOESM1_ESM.docx]
