# Supplementary material for: Plant Screen Mobile: an open-source mobile device app for plant trait analysis
Source: Plant Methods. 2019 Jan 11;15:2. doi: 10.1186/s13007-019-0386-z (PMC6329080; doi:10.1186/s13007-019-0386-z)
Supplement: Supplementary file 2 — Additional file 2. Application Scenarios for Plant Screen Mobile. [file 13007_2019_386_MOESM2_ESM.docx]

**Additional file 2: Application Scenarios for Plant Screen Mobile**


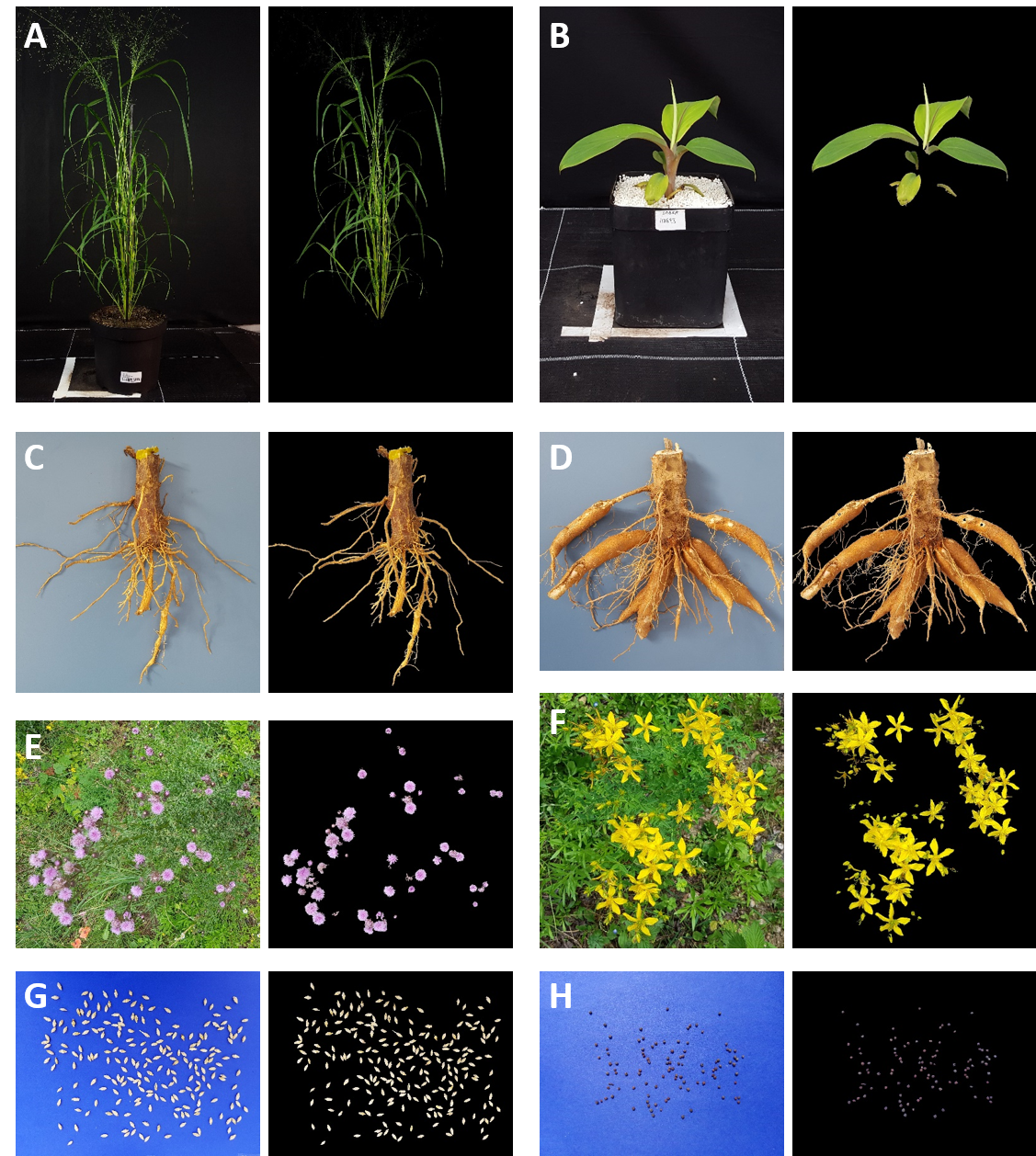


With the following examples we want to illustrate different application scenarios for PSM, where a proper segmentation is needed to compute further plant traits. All examples show the original input RGB and the respective segmentations of the desired target. Each example was processed with a HSV segmentation. The first 2 examples display typical images from our study, (A) - *Eragrostis tef* and (B) - banana. Images were taken in a lab setup with black background and controlled illumination, which facilitates the segmentation process. The next 2 examples in (C) and (D) display Cassava roots before and during storage root formation. Also here we facilitated the segmentation by using a bluish background. Such segmentations can be used to estimate root biomass, width and length of the root system and thickness of storage roots. (E) and (F) show the segmentation of blossoms in front of a green plant background. No artificial background could be applied here, but the color properties of the image allowed for an easy segmentation. Segmentation of blossoms (or flower buds) and subsequent counting can e.g. be used as a proxy for yield estimation. The last two examples in (G) and (H) show that PSM can also be used for seed segmentation and seed counting. In this case we used a proper background and distributed (G) – barley and (H) - rape seeds over a blue paper.
